# Determination of excited state dipole moments in solution via thermochromic methods

**DOI:** 10.1016/j.mex.2020.101101

**Published:** 2020-10-18

**Authors:** Mirko Matthias Lindic, Matthias Zajonz, Marie-Luise Hebestreit, Michael Schneider, W. Leo Meerts, Michael Schmitt

**Affiliations:** a Heinrich-Heine-Universität, Institut für Physikalische Chemie I, D-40225 Düsseldorf, Germany; b Radboud University, Institute for Molecules and Materials, Felix Laboratory, Toernooiveld 7c, 6525 ED Nijmegen, the Netherlands

**Keywords:** Thermochromic methods, Thermochromic shifts, Excited state dipole moments, Spectroscopy, Fluorescence, Solvent polarity, Anisole, Absorbance, Density measurements, Cavity volume

## Abstract

The method basically combines the existing ideas of excited state dipole moment determination via thermochromic fluorescence spectroscopy with the determination of the solvent cavity volume via concentration dependent density measurements of the solution densities at different weight fractions. Additionally, the determination of the cavity volume in dependence of the solvent temperature is included here, which provides a better accuracy of the excited state dipole moment determination. With this step two major sources of errors are eliminated: the use of the very imprecise Onsager radius and the assumption, that the cavity size is temperature independent.

• Thermochromic absorption and fluorescence spectroscopy.
• Cavity volume determination by density measurements.
• Temperature dependent cavity volume determination.

## Graphical abstract

**Figure:**
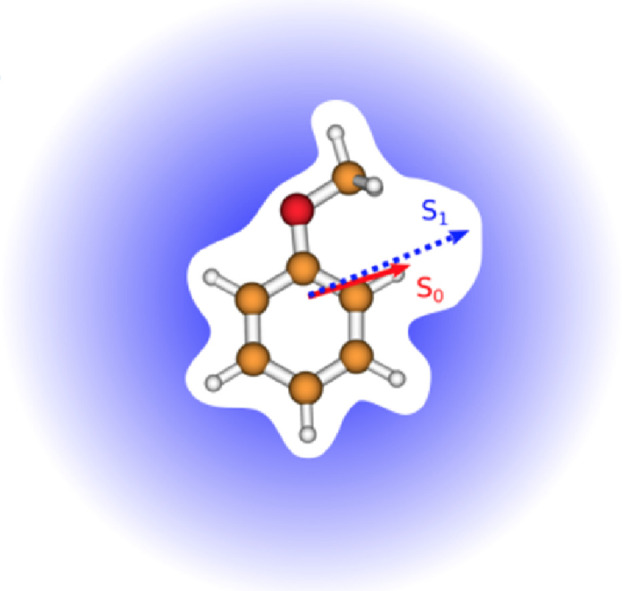

**Table:** Specifications Table

Subject Area	*Chemistry*
More specific subject area:	*Molecular Spectroscopy* *Physical Chemistry*
Method name:	*Thermochromic absorption and fluorescence spectroscopy with advanced solvent effect approximation*
Name and reference of original method:	*•* M.M. Lindic, M. Zajonz, M.-L. Hebestreit, M. Schneider, W. L. Meerts, and M. Schmitt, *J. Photochem. Photobiol. A: Chem.* (2018) 365, 213–219 *• E. G. Demissie, E. T. Mengesha and G. W. Woyessa, J. Photochem. and Photobiol. A, 2016, 337, 184–191* *• A. Kawski, B. Kuklinski and P. Bojarski, Chem. Phys. Letters, 2005, 415, 251–255*
Resource availability:	*M. M. Lindic, M. Zajonz, M.*-*L. Hebestreit, M. Schneider, W. L. Meerts and M. Schmitt, Data in Brief 21 (2018) 313–315*

## Method details

This section will give a step-by-step instruction for performing the method of thermochromic shifts for determination of excited state dipole moments.

## Introduction

Although the determination of excited state dipole moments using solvatochromic shifts of absorption/emission spectra in different solvents has many shortcomings, that are well-documented [1], [2], [3], [4]–5], it is still widely applied experimentally [6], [7], [8]–9] and investigated theoretically [10], due to its conceptual and experimental simplicity. Recently, dipole moment changes upon electronic excitation, even of different conformers, could be resolved in solution by a combination of stationary and time-resolved fluorescence measurements and ultraviolet−visible transient absorption spectroscopy [11]. A recent review on excited state dipole moments compares the different methods that are used for the determination of dipole moments of molecules in their electronically excited states [12]. These methods comprise solvatochromism [13], electrochromism [14], thermochromism [15] (in solution) and direct Stark effect measurements [16,17] (in the gas phase).

Some of the problems that are connected with the solvatochromic method can be solved, if one makes use of the fact, that variation of the solvent polarity function might as well be reached by variation of the temperature of the solution in spite of the solvent. This is due to the fact that both index of refraction and permittivity, which make up the solvent polarity function, depend on the temperature of the solvent. However, spectral shifts, which are induced by temperature variations are smaller than those, originating from variation of the solvent. Therefore, a good temperature stability and a reproducible determination of absorption and emission maxima are crucial for the success of the method. One of the major problems of the methods of solvatochromic shifts, the differing interactions of the solute with different solvents can be avoided by the use of thermochromic shifts. Apart from the use of thermochromic shifts in spite of solvatochromic shifts, replacement of the original Onsager radius [18] for description of the cavity size by the experimentally determined cavity volume greatly improves the method. In the following we describe, how the method can be employed reliably.

We have chosen two different systems to describe the method in detail. Anisole [19,20] and 2-((4-methoxyphenyl)ethynyl)-3-(1-methyl-1H-indol-3-yl)quinoxaline (Q1) [21], both in ethyl acetate solution have been chosen as examples for medium size dipole moment and dipole moment change (anisole) and large dipole moment and dipole moment change upon excitation for Q1.

## Density measurement

Accurate density measurements of the solution as function of the temperature have been performed, using a high precision density meter with temperature control (Anton Paar DMA4500M). Concentration (weight fraction) dependent density measurements in a wide temperature range are necessary. The weight fractions are chosen to match the accuracy of the density meter, but should be as low as possible for the concentration used in spectral measurements. Representative results can be produced using eleven different weight fractions between 0.0013 and 0.0129 for anisole and between 0.0004 and 0.004 for Q1 and temperatures between 263 K and 343 K with an increment of 2 K. To minimize concentration fluctuation and maximize precision, the measurements are performed starting at low temperatures.

## Evaluation of the cavity volume from density measurements

According to Eqs. (1) and (2) the cavity volume is calculated from the slope of the linear regression of the inverse density versus weight fraction *w* of the solute at each temperature.

(1) $$\frac{1}{\rho }=\frac{1}{\rho^{*}}+\left(\frac{V_{m}}{M_{w}}-\frac{1}{\rho^{*}}\right)\cdot w$$

(2) $$V_{m}=\left(S+\frac{1}{\rho^{*}}\right)\cdot M_{w}$$

**Fig. 1:**
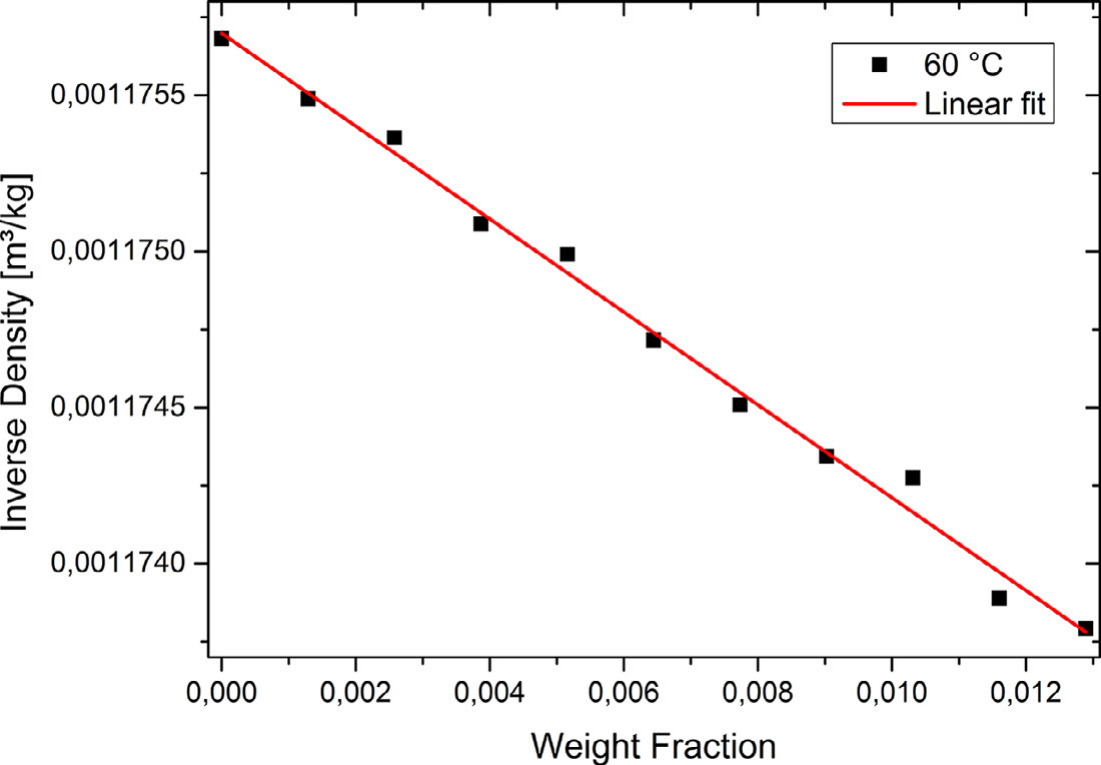
Plot and fit of inverse density vs. weight fraction of anisole in ethyl acetate at 333 K.

Here, ρis the actual density of the solution, $\rho^{*}$is the density of the pure solvent, $V_{m}$is the molar cavity volume, $M_{w}$is the molecular weight, wis the weight fraction and Sis the slope of the linear regression of 1/ρvs. *w*. An example diagram at a temperature of 60 °C including the linear fit is given in Fig. 1.

**Fig. 2:**
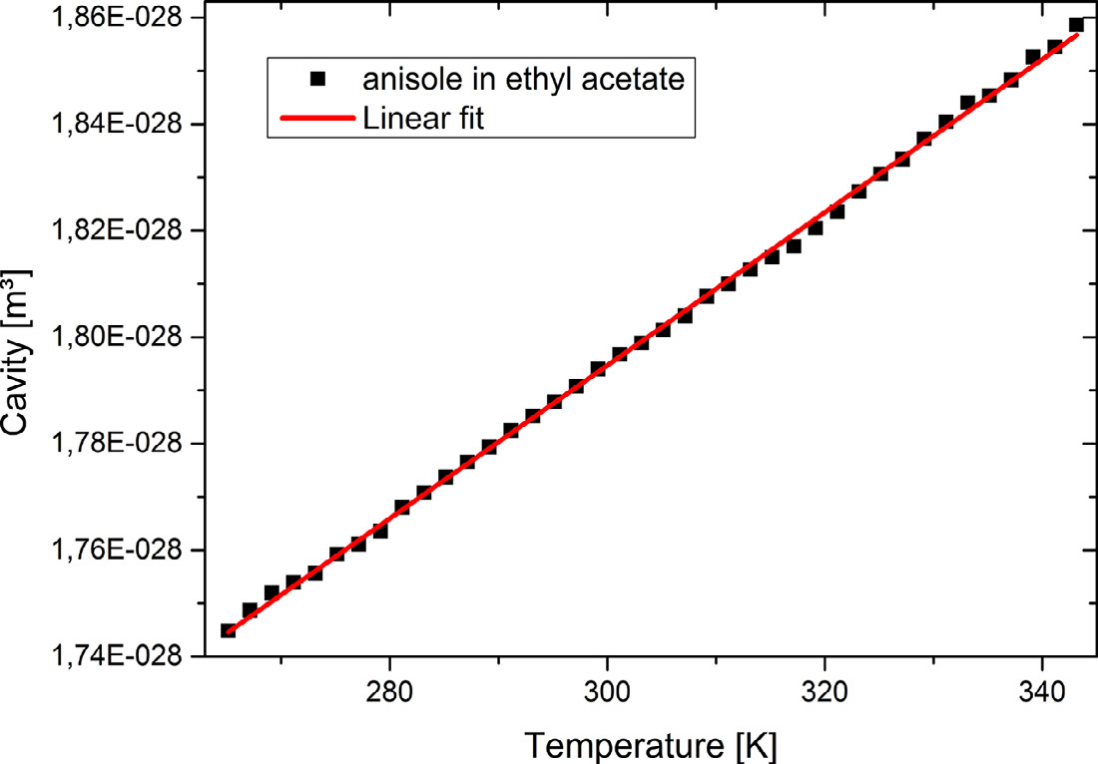
Plot and fit of cavity volume versus temperature for anisole in ethyl acetate.

From the measured cavity volume at each temperature the function V(T)is generated, which is the linear regression of the molecular cavity volume versus the temperature, shown in Fig. 2.

## Permittivity measurements

The permittivity of the solvent which is needed for the solvent polarity function, has to be determined in a temperature range, as large as possible. To provide precise measurements, the capacity of a liquid test fixture (Keysight 16452A) is measured with an E4990A impedance analyzer. Temperature dependent measurements are performed by placing the fixture in an oil tank, which is temperature controlled via an external liquid cooling.

For determination of the permittivity of a solvent its capacity is measured in a temperature range between 277 K and 323 K with intervals of 2 K using the Keysight 16452A liquid test fixture. Finally, the measured capacity is divided by the air capacity of the capacitor according to Eq. (3).

(3) $$\varepsilon_{r}=\frac{C_{p}}{C_{p}^{0}}$$

**Fig. 3:**
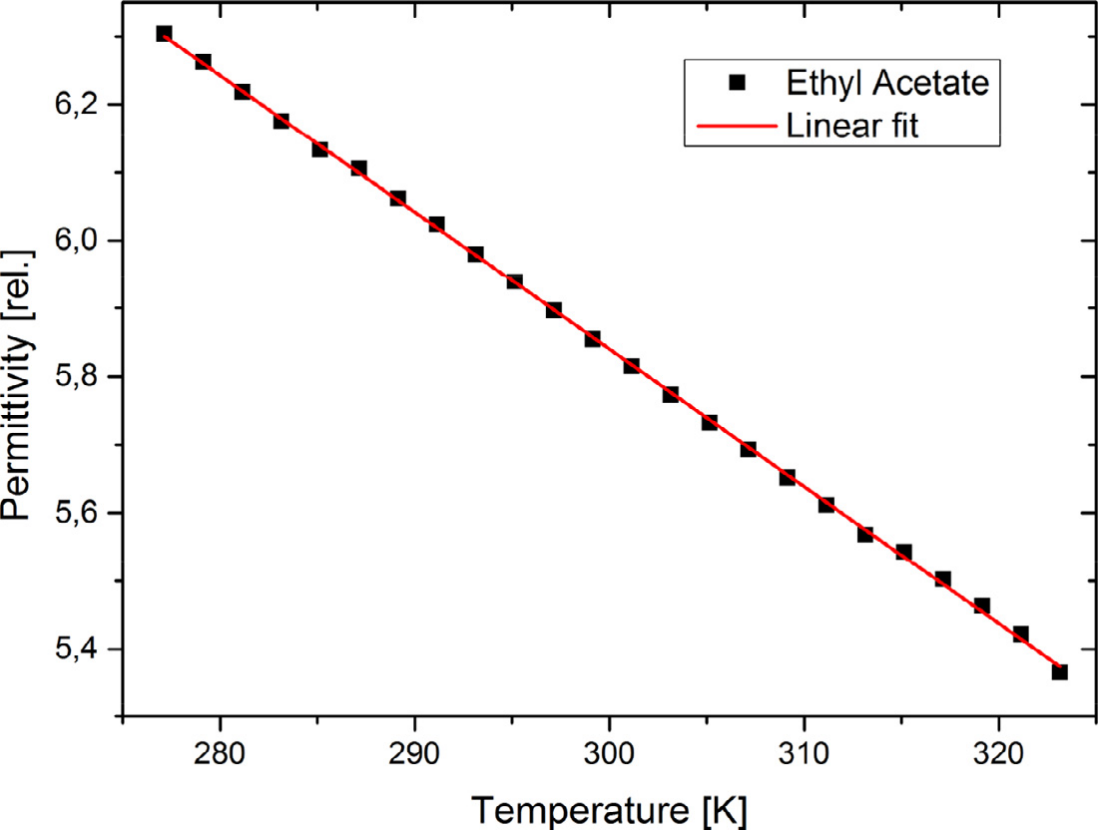
Temperature dependent permittivity of ethyl acetate including linear fit of the measurement data.

Fig. 3 shows the temperature dependent permittivity of ethyl acetate in a temperature range of 277 K to 323 K. The data were fitted to a linear function which can be extrapolated for later evaluation.

## Determination of the index of refraction

**Fig. 4:**
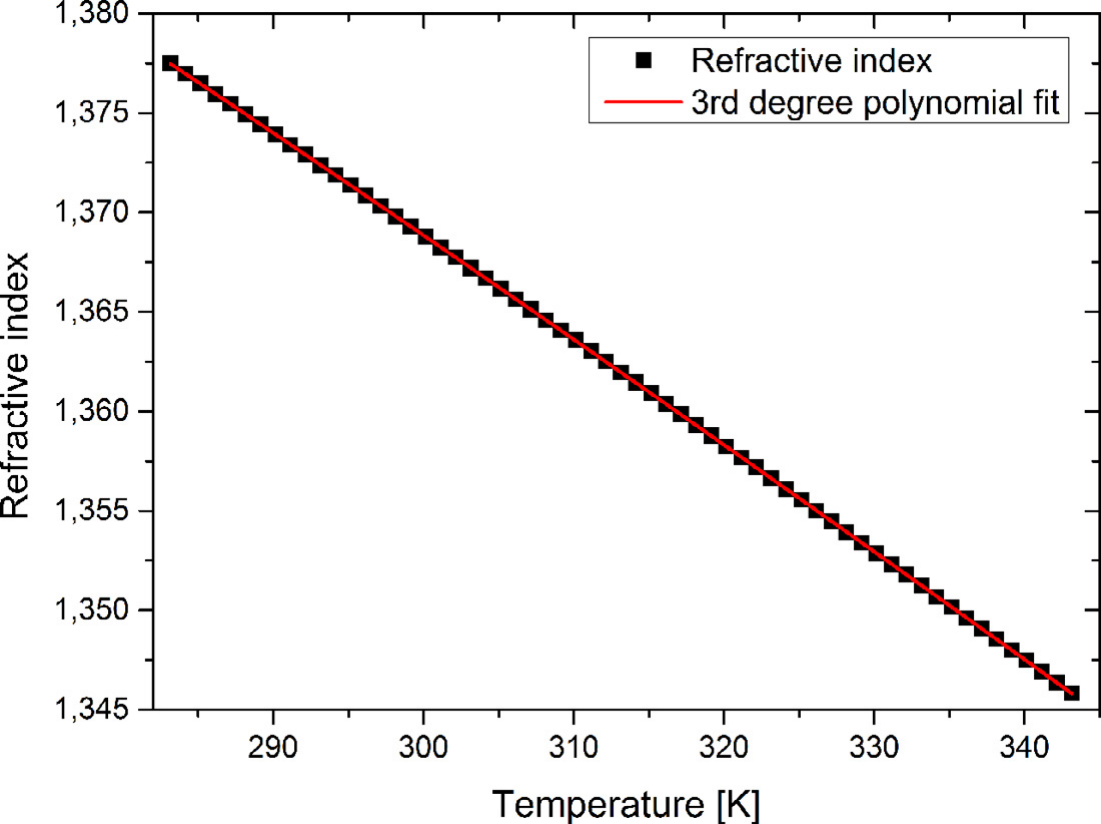
Temperature dependent index of refraction of ethyl acetate including polynomial fit.

Additionally to the temperature dependent cavity volume and the temperature dependent permittivity, the temperature dependent index of refraction of the chosen solvent is essential to calculate the solvent polarity function. For precise measurements of the temperature dependent index of refraction, we used an Anton Paar Abbemat MW refractometer, which provides good temperature control in a range of 283 K to 343 K. Fig. 4 shows the values of the index of refraction of ethyl acetate at a wavelength of 589.6 nm including a third order polynomial fit of the data.

## Determination of the ground state dipole moment from permittivity measurements

### Spectral measurements

**Fig. 5:**
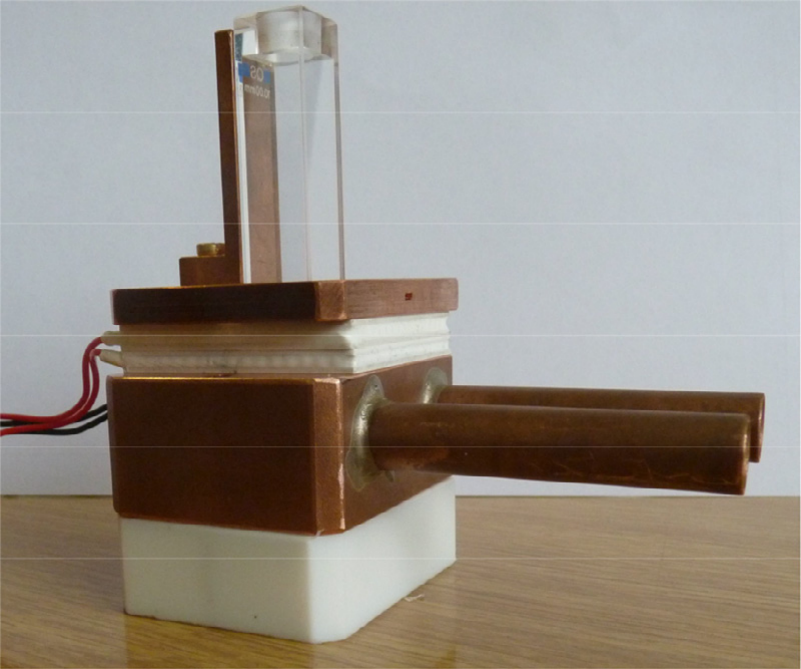
Cooling and heating unit, including cuvette, Peltier elements and liquid cooling inside of copper block.

The absorption and fluorescence spectra should be measured in a large temperature range within the limits given by freezing and boiling points of solvent and to provide better evaluation the interval should be about 2 K. For measurements below 273 K, water vapor condensation has to be prevented on the transmitting surfaces. In order to minimize temperature fluctuations and condensation on the surface, a vacuum chamber was built around the temperature controlled parts of the apparatus. The vacuum chamber contains the cuvette, which is insulated to the lid of the chamber on the top and placed inside a copper frame which conducts the heat to a double-stage Peltier unit. It's hot side is cooled by a liquid cooling cycle. The set-up is shown in Fig. 5.

**Fig. 6:**
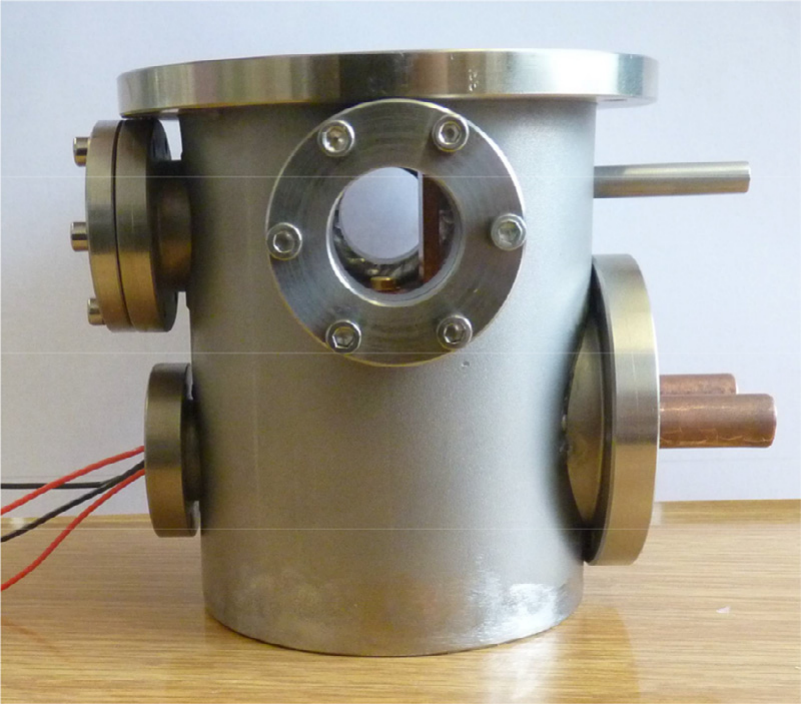
Housing of the spectroscopy chamber with view through the transmission spectroscopy windows.

**Fig. 7:**
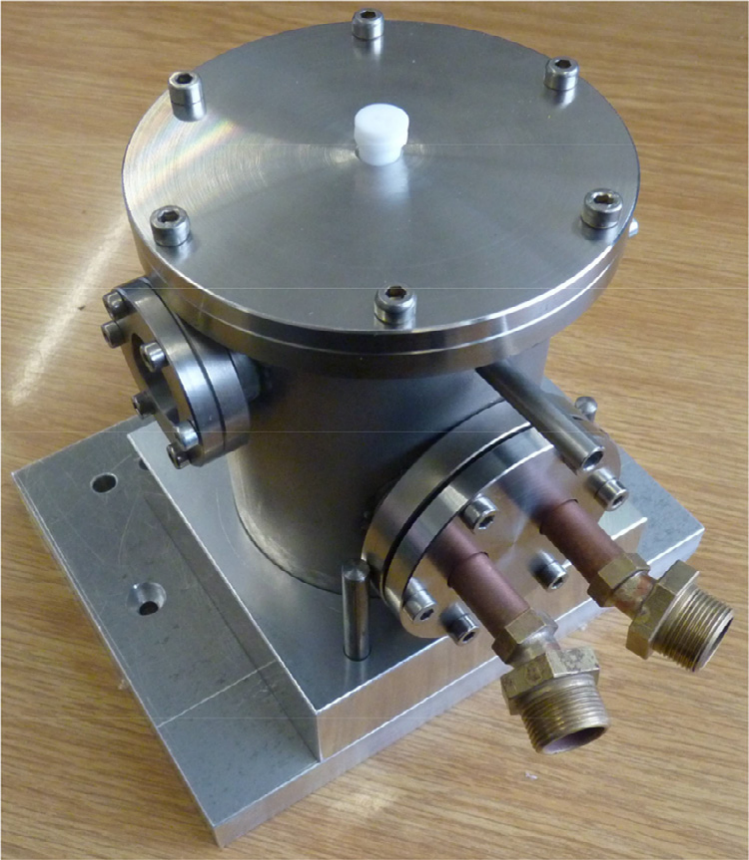
Housing of the measurement chamber with closed lid and liquid cooling connectors. The sample can be manipulated without breaking the vacuum.

The stainless-steel vacuum chamber is supplied with feedthroughs for the liquid cooling and the electric connections as well as with three Suprasil I windows for light transmission. The lid of the chamber gives direct access to the cuvette for an easy handling of the sample without necessity to break the vacuum. Two perspectives of the chambers outside are given in Figs. 6 and 7.

The vacuum chamber is equipped with an adapter to fit it both in the absorption as well as in the fluorescence emission spectrometer. As in the density measurements, the data are collected, starting from the lowest temperature and heating up the sample step by step. A PID control of the Peltier unit is used to stabilize the temperature during the measurement of the spectra to an accuracy better than 0.1 K.

### Evaluation of the spectral shifts

**Fig. 8:**
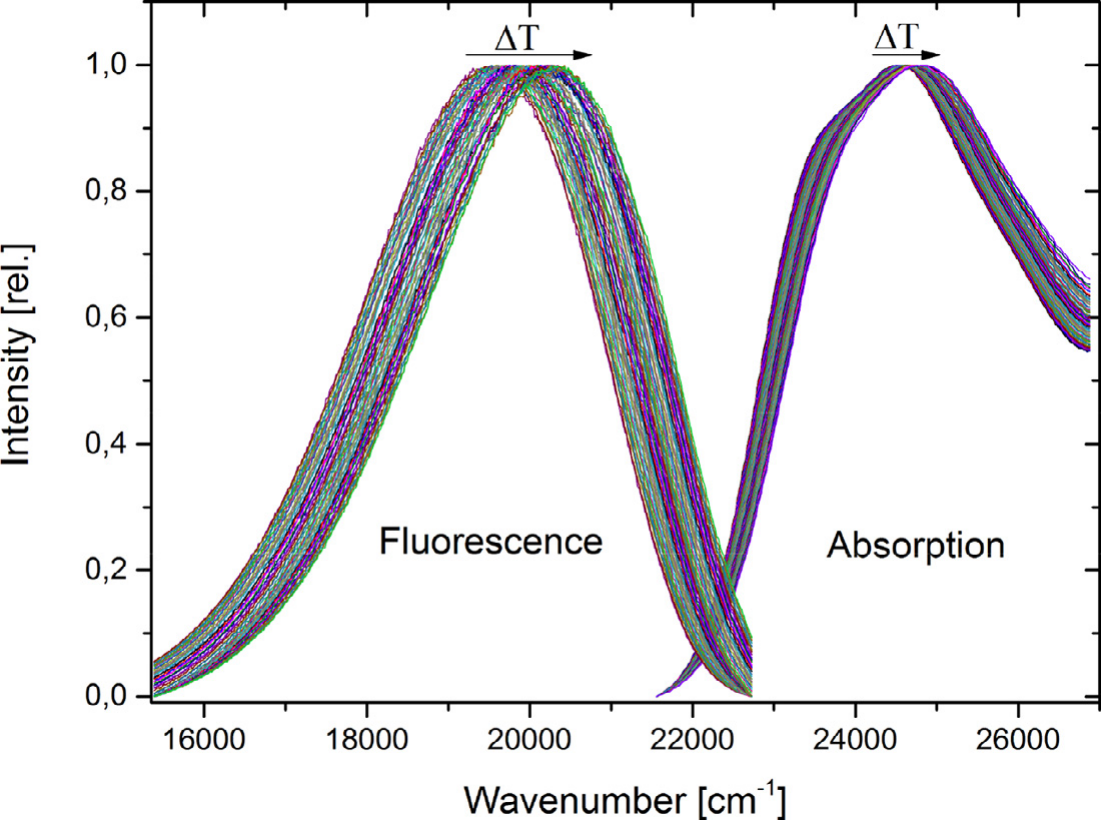
Absorption and emission spectra of Q1, measured is ethyl acetate. Temperature shift is indicated by text and arrows. Spectra are normalized but not fitted.

**Fig. 9:**
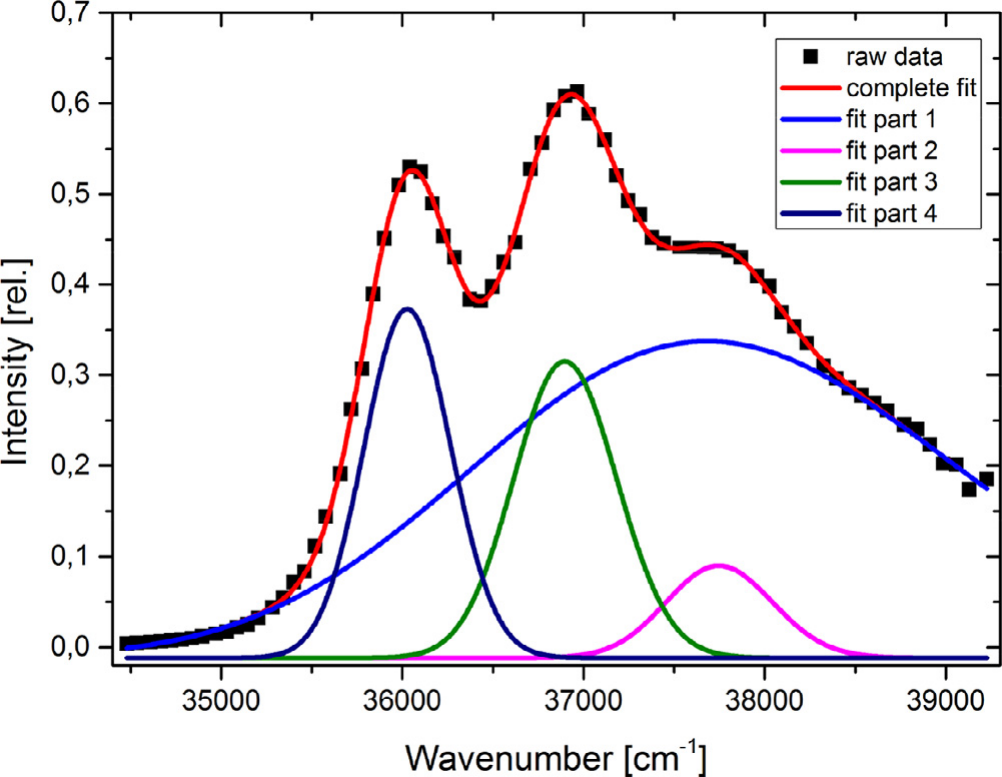
Construction of a fit-function (red trace) consisting of 4 Gauss-functions applied to the raw absorption spectrum of anisole in ethyl acetate at 293.15 K.

**Fig. 10:**
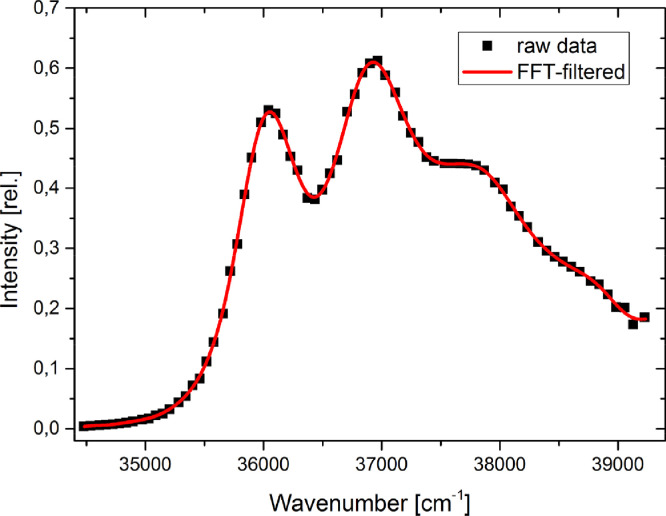
Absorption spectrum of anisole (black squares) and the smoothened function (red trace) using a low bandpass Fourier filter with a cut-off frequency of 0.35 nm^−1^.

Since thermochromic shifts are smaller, than solvatochromic shifts, the evaluation of the maximum of the absorption and emission bands is crucial. The unfitted absorption and fluorescence spectra of Q1 are shown in Fig. 8, additional data for anisole can be found in [8,9]. It is necessary to apply a good fit to the measured data, to avoid measurement artifacts or noise that could lead to an artificially shifted band. The best results have been found using either a fit function, which is constructed from multiple Gauss functions or a fast Fourier transformation (FFT) filter to the raw data. Both methods can give good results, but the FFT-Filter is easier to apply. Both methods are described in the following. The maximum position of the spectra can be determined from fits to a sum of several Gauss functions, whose number have to be determined manually. Examples for the fit-function applied to the data and the construction of one fit-function are given in Fig. 9 . The maxima of the functions can either be found with a peak-picking tool or by using the fit-function and finding the analytical solution. For the evaluation, the longest wavelength maximum of the absorption spectrum and the overall maximum of the fluorescence spectrum is needed. The second method for determination of the maximum of the intensities in the absorption and emission spectra is the usage of a Fast Fourier Transformation (FFT) Filter. With this method the high frequency noise is eliminated and a smoothened graph without change in positions and intensities is obtained. An example of FFT-filtered maximum of the absorption is given in Fig. 10 .

For anisole, both of the fit methods agreed within 0.2 cm^−1^. However, for a different molecule, 1-methylindole, we found a mean deviation of about 9 cm^−1^ but a maximum deviation of more than 100 cm^−1^ for both methods, which is a large difference. The overall influence of the fit on the final dipole moment results in a deviation of 0.15 D, which is, regarding the accuracy of the method, not a big difference. On the other hand, the precision of the method can in no case be better, more likely be worse, than 0.15 D, even if the statistical error may be smaller.

### Evaluation of the dipole moment

First, the solvent polarity functions of Lippert-Mataga [22,23] and Bilot-Kawski [24,25], given in Eqs. (3) and (4), which include the experimentally determined cavity volume function V(T), refractive index n(T)data and permittivity ε(T)of the solvent have to be determined.

(3) $$F_{LM}\left(T\right)=\frac{1}{V\left(T\right)}\left[\frac{\varepsilon \left(T\right)-1}{2\varepsilon \left(T\right)+1}-\frac{1}{2}\left(\frac{n{\left(T\right)}^{2}-1}{2n{\left(T\right)}^{2}+1}\right)\right]$$

(4) $$F_{BK}\left(T\right)=\frac{1}{V\left(T\right)}\left(\frac{2n{\left(T\right)}^{2}+1}{n{\left(T\right)}^{2}+2}\left(\frac{\varepsilon \left(T\right)-1}{\varepsilon \left(T\right)+1}-\frac{n{\left(T\right)}^{2}-1}{n{\left(T\right)}^{2}+2}\right)+\frac{3\left(n{\left(T\right)}^{4}-1\right)}{{\left(n{\left(T\right)}^{2}+2\right)}^{2}}\right)$$

Plots of the sum and of the difference of the wavenumber maxima vs. the different solvent polarity functions, according to (5), (6), (7) have to be prepared. For Eq. (7) which was introduced by Demissie et al. [26], it is possible to apply several solvent polarity functions *F_X_* for the evaluation.

(5) $${\tilde{\nu }}_{A}\left(T\right)+{\tilde{\nu }}_{F}\left(T\right)=-\frac{2\left(\mu_{e}^{2}-\mu_{g}^{2}\right)}{3\varepsilon_{0}hc}\cdot \;F_{BK}\left(T\right)+const.$$

(6) $${\tilde{\nu }}_{A}\left(T\right)-{\tilde{\nu }}_{E}\left(T\right)=\frac{2{\left(\mu_{e}-\mu_{g}\right)}^{2}}{3\varepsilon_{0}hc}\cdot \;F_{LM}\left(T\right)+const.$$

(7) $${\tilde{\nu }}_{A/E}\left(T\right)={\tilde{\nu }}_{A/E}^{0}-\frac{2\mu_{g/e}\left(\mu_{e}-\mu_{g}\right)}{3\varepsilon_{0}hc}\cdot \;F_{X}\left(T\right)$$

From the slope of the linear regression of the data, the dipole moments can be calculated according to (8), (9), (10), (11).

(8) $$\mu_{e}=\sqrt{\mu_{g}^{2}+\frac{3m_{BK}\varepsilon_{0}hc}{2}}$$

(9) $$\Delta \mu =\sqrt{\frac{3m_{LM}\varepsilon_{0}hc}{2}}$$

(10) $$\mu_{g}=\sqrt{\frac{3\varepsilon_{0}hcm_{A}^{2}}{2\left(m_{E}-m_{A}\right)}}$$

(11) $$\mu_{e}=\sqrt{\frac{3\varepsilon_{0}hcm_{E}^{2}}{2\left(m_{E}-m_{A}\right)}}$$

**Fig. 11:**
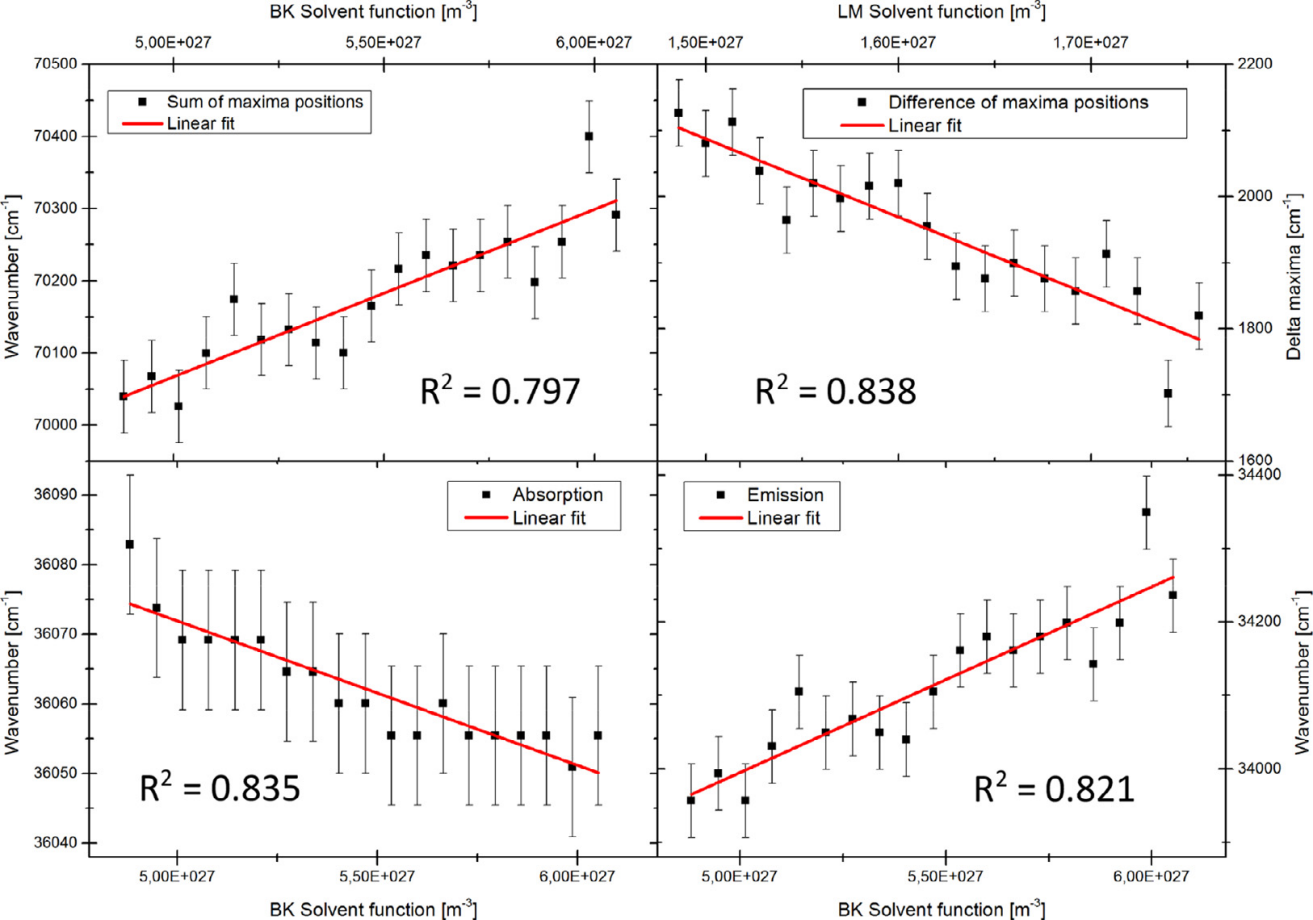
Plots of maxima wavenumbers versus solvent polarity functions of Bilot-Kawski and Lippert-Mataga. The sample molecule is anisole with solvent ethyl acetate. *R*^2^ for the goodness of the individual fits is shown in each graph.

**Fig. 12:**
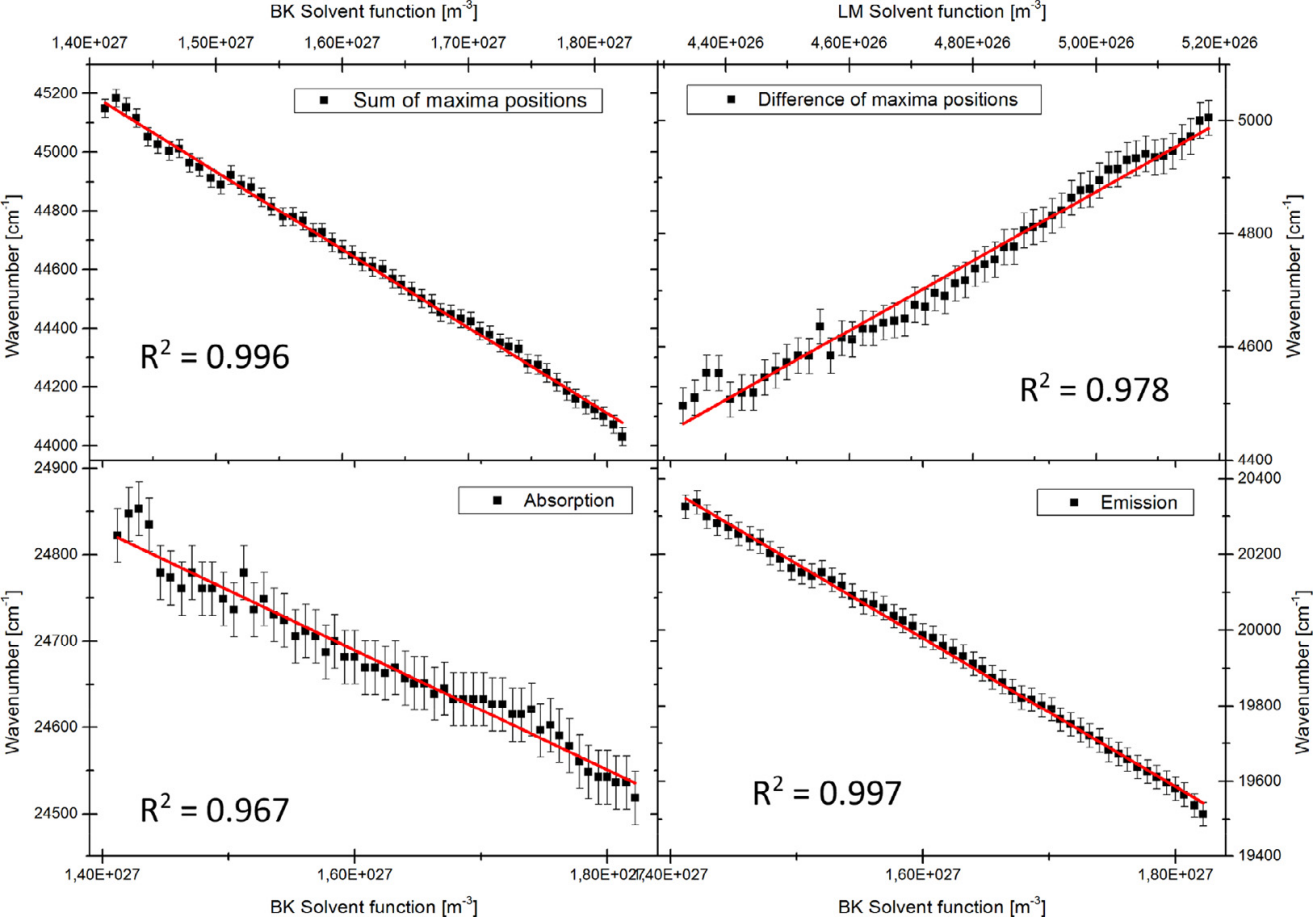
Plots of maxima's wavenumbers versus solvent polarity functions of Bilot-Kawski and Lippert-Mataga. The sample molecule is 2-((4-methoxyphenyl)ethynyl)-3-(1-methyl-1H-indol-3-yl)quinoxaline with solvent ethyl acetate. *R*^2^ for the goodness of the individual fits is shown in each graph.

Here, $m_{x}$(*x = LM, BK, A, E*) are the slopes of the diagrams according to (5), (6), (7) , respectively, $\varepsilon_{0}$is the permittivity of the vacuum, his Planck's constant, cis light speed in vacuum and $\mu_{x}$(*x = g, e*) are the dipole moments of ground (*g*) and excited state (*e*). Examples for plots, according to (5), (6), (7) , are shown in Figs. 11 and 12.

Since the dipole moment changes upon excitation are larger for Q1 compared to anisole, the fits perform better for the former and show a much better *R*^2^. The resulting changes of dipole moments of anisole and Q1 upon electronic excitation from the plots of Figs. 11 and 12 and (8), (9), (10), (11) are summarized in Tab. 1 along with calculated dipole moment changes from *ab initio* calculations and the gas phase value (for anisole) from rotationally resolved electronic Stark spectroscopy [19].

**Table 1:** Changes of the permanent dipole moments of anisole and Q1 upon electronic excitation in Debye according to the evaluations discussed in the main text. Although Demissie's method allows for an independent determination of the ground state dipole moment, we evaluated the change of dipole moment for anisole using the much more exact value from microwave Stark spectroscopy [27].

	Bilot-Kawski	Demissie (BK)	Demissie (LM)	Lippert-Mataga	CC2/cc-pVTZ	Gas phase
Anisole	1.5(3)	1.2(3)	3.2(6)	5.4(4)	0.97	0.93(1)
Q1	5.27(3)	5.49(11)	8.3(5)	12.1(2)	6.48	–

Considering the large difference of both molecular systems, we see a quite uniform pattern emerging: Evaluation of the dipole moment changes using Bilot-Kawski's or Demissie's methods yields good results if the Bilot-Kawski solvent polarity function is used. Evaluation of dipole moment changes using Lippert-Mataga's plots, either in the original version, or as solvent polarity function in Demissie's method exaggerates the dipole moment change considerably. The remaining discrepancies between the results from the referencing methods, CC2/cc-pVTZ and gas phase measurement, and the results from thermochromic spectroscopy can be traced back to the expectable change of the dipole angle during electronic excitation. The change of the dipole orientation is by now not considered in the applied theories.

However, measuring thermochromic absorption and emission spectra, combined with exact determination of all necessary temperature dependent parameters, yields results, which show a clean linear trend with small deviations. Therefore, a reasonable evaluation can be facilitated for the dipole moments, depending on the used evaluation method. By comparison to linear plots from solvatochromic measurements from the literature the improvement of the dipole determination from thermochromic rather than solvatochromic shifts is obvious. Also the accuracy of results, compared with *ab initio* calculations or reasonable reference methods, is increased compared to different works from the past.

Supplementary material *and/or* Additional information:

M. M. Lindic, M. Zajonz, M.-L. Hebestreit, M. Schneider, W. L. Meerts and M. Schmitt, Data in Brief 21 (2018) 313–315
